# The association of the level of self-care on adherence to treatment in patients diagnosed with type 2 diabetes

**DOI:** 10.1007/s00592-020-01628-z

**Published:** 2020-11-29

**Authors:** Sylwia Krzemińska, Katarzyna Lomper, Anna Chudiak, Davide Ausili, Izabella Uchmanowicz

**Affiliations:** 1 Department of Clinical Nursing, Faculty of Health Sciences, Wroclaw Medical University, Wroclaw, Poland; 2 Department of Medicine and Surgery, University of Milano-Bicocca, Monza, Italy

**Keywords:** Nursing, Type 2 diabetes, Self-care, Adherence to treatment, Self-care of diabetes index (SCODI), Adherence in chronic diseases scale (ACDS)

## Abstract

**Aims:**

The study aimed to assess the impact of self-care on adherence to treatment in patients diagnosed with type 2 diabetes and effect of complex interaction of social, lifestyle, economic, environmental and behavioural.

**Methods:**

The study was carried out between June 2018 and May 2019 on 324 patients (162 females, 162 males) with type 2 diabetes. To measure the levels of self-care, the Self-Care of Diabetes Index (SCODI) questionnaire was used. Adherence to treatment was assessed with the Adherence in Chronic Diseases Scale (ACDS).

**Results:**

The highest scores of health behaviour were on the subscale of adherence with the mean value of 68.37, and the lowest results on the subscale of blood sugar self-monitoring, with the mean of 56.05. We found that low adherence to treatment was present in 52.47% of respondents, the moderate level in 39.20%, while only 8.33% of patients showed the high level. There were significant positive correlations between the ACDS and SCODI subscales (*p* < 0.05): self-care maintenance (0.436), self-care management (0.413), self-care monitoring (0.384), and self-care confidence (0.453).

**Conclusions:**

Self-care affects on adherence in patients with type 2 diabetes. The higher self-efficacy in each of the areas of functioning, the higher the level of adherence to treatment. We found that demographic variables such as female sex, education and employment status can influence self-care in managing chronic illnesses such as type 2 diabetes.

## Introduction

The epidemiological data show a significant worldwide growth of people with diabetes melitus (DM), which is currently one of the leading causes of global mortality [1]. In addition to genetic predispositions, it is important to stress the importance of the so-called modifiable factors, such as a sedentary lifestyle and unhealthy diet, which are significant predictors of the growing diabetes epidemic [2]. The disease has grown into a major public healthcare problem. The research on the subject reveals that in 2017 there were 425 million people with DM, and this number will more likely rise to 693 million by 2045 [3].

Uncontrolled (out of the therapeutic range) DM and chronic hyperglycaemia are a cause of severe health conditions mainly associated with damaging the vascular system but may also impair eyesight, nerves, kidneys and the heart [4]. Regular check-ups and follow up of patients diagnosed with DM may help prevent or delay health complications. Long-term measures present a great challenge to healthcare workers, as the needs of patients as regards self-care include not only regular blood glucose testing but also measures aimed at preventing the complications of the disease, including disability. Self-monitoring, which is referred to in the literature as diabetes-related self-care activities (DRSCAs), is a key element in the management of DM. DRSCAs relate to the active and effective self-management of the condition by the patient. Self-monitoring is a strategy focusing primarily on self-organisation of patient’s life to ensure adherence to medication but also to monitor meals, blood glucose monitoring, physical activity and the practice of self-care routines, e.g. feet care [5].

American Diabetes Association (ADA) [6] stresses the importance of health education and self-care as a key element in the management of diabetic patients. Health education is of particular importance, as patients with type 2 DM face numerous challenges like regular check-ups, adherence to treatment and self-care. An adequate health education is associated with good metabolic control and helps to prevent of compliactions associated with DM [7]. The main goal of health education among patients with DM is to achieve changes in health behaviours [8]. However, patients often find self-management difficult to engage in the treatment process on a daily basis, especially regarding daily blood glucose monitoring. As many as 85.7% of DM patients fail to achieve therapeutic haemoglobin A1C (HbA1c) levels [6].

The abnormal blood glucose levels in DM patient population may result from wrong nutrition habits, poor physical activity and non-adherence to treatment [9]. The available literature indicates many factors influencing the phenomenon of non-adherence in patients with diagnosed DM. Several studies carried out to date have discovered that education and economic status are key determinants of adherence in the population discussed. For instance, patients who are unemployed, younger and those with tertiary education are more likely to adhere to treatment [10]. The additional factors that may have a negative influence on blood glucose levels in DM patients also include insufficient knowledge, low self-esteem and none or poor social and family support. Recent study proves that a patient’s family is also important in supporting patient’s self-management behaviours [11]. Obviously, the literature reports other multiple factors which have an influence on adherence to treatment and the level of self-care in diabetes patients. For instance, promoting health behaviours by clinicians and healthcare workers is out of questioning.

Recent research findings on patients with chronic conditions suggest to take multifaceted efforts in order to improve adherence and self-care in this group of patients [12]. DM self-care involves not only daily blood glucose testing and adherence to medication but also modification of lifestyle, including diet, as well as a number of other measures, which should be supported by medical personnel. Indeed, effective management of DM is a difficult task for patients who very often do not understand the bottom line of self-care [13].

The aim of this study was to assess the impact of self-care on adherence to treatment in patients diagnosed with type 2 DM and effect of complex interaction of social, lifestyle, economic, environmental and behavioural.

## Methods

### Design and participants

This observational and correlational study was carried out between June 2018 and May 2019 at the Internal Medicine Department of Hospital in Trzebnica (Poland), and the Diabetes Unit of Outpatient Clinic in Wroclaw (Poland). The STROBE guidelines (Strengthening the Reporting of Observational Studies in Epidemiology) were followed.

The study included 180 patients with diagnosed type 2 diabetes from each centre (360 patients in total). Participants included in the study met the following criteria for inclusion: type 2 DM, over 18 years of age, duration of illness of over 1 year, lack of cognitive disorders, ability to complete questionnaires on one’s own, and patient’s consent to take part in the study.

Due to incompleteness of research questionnaires or resignation from participation in the study finally, a group of 324 patients (162 females, 162 males) with type 2 DM was involved into analysis. Duration of illness was as follows: up to 5 years for 123 patients (37.96%), 5–10 years for 80 (24.69%), over 10 years for 120 (37.04%), and no data available in case of 0.31% patients.

### Research tools

In order to assess the level of self-care, the Self-Care of Diabetes Index (SCODI) questionnaire was used to measure self-care in DM patients. The questionnaire assesses the self-efficacy of a diabetes patient in four areas of daily living: self-care maintenance, self-care monitoring, self-care management and self-care confidence. Respondents’ scores range from 0 to 100, with the higher score indicating the higher level of self-efficacy in each domain of self-care behaviours. However, there are no available standards as to what scores indicate a high level of self-efficacy and what scores indicate a low level of self-efficacy. As all the areas are scored on the same scale, it is possible to compare the level of self-efficacy in different areas in order to identify any self-care deficits [14]. The study used the Polish adaptation of the tool. The SCODI questionnaire has acceptable internal consistency and reliability in assessing self-care among diabetic patients in the Polish population with Cronbach’s Alpha: self-care maintenance (0.759), self-care monitoring (0.741), self-care management (0.695) and self-care confidence (0.932) [15].

The level of adherence to treatment was assessed with the Adherence in Chronic Diseases Scale (ACDS), which is designed for the assessment of patients with chronic conditions. The questionnaire contains 7 questions. Questions 1–5 address behaviours directly influencing adherence to treatment, whereas questions 6–7 examine/test patients’ convictions and situations which have an indirect influence on adherence. The possible score range is 0 to 28—the higher the score, the higher the level of adherence to treatment [16].

**Ethical considerations**

The study was approved by the Wroclaw Medical University Bioethics Committee (approval no. KB–622/2018). All patients provided informed consent, and were informed that they could withdraw from the study at any time. The study was carried out in accordance with the tenets of the Declaration of Helsinki and Good Clinical Practice guidelines.

### Statistical analyses

A multiple factor analysis of the effect of independent variables was carried out using linear regression. The results were presented as the values of the regression model parameters with a 95% confidence interval. A significance level of 0.05 was used in the analysis. Thus, all *p* values of less than 0.05 were interpreted as indicating a strong correlation. To assess how well the model fits the data, we used the R-squared determination coefficient. This measure is the proportion of the variance in the depedent variable that is explained by the model. The analysis was carried out using the R software, version 3.6.0 [17].

## Results

### Sociodemographic and clinical analysis

The study was performed on 324 patients (162 females, 162 males). Over half of the respondents were aged over 60 (54.01%). The largest group of respondents were those who had been diagnosed up to 5 years before (37.96%), followed by those who had been diagnosed over 10 years before the study (37.04%). As regards the place of residence, 68.83% of the respondents lived in urban areas and 30.56% lived in rural areas. As for the level of education, 34.57% of the respondents had secondary education, 34.26% vocational education, 17.9% tertiary education and 13.27% primary education. Of the respondents, 37.96% were retired, 37.35% declared that they were employed, 13.58% were retired on ill-health grounds and 11.11% were unemployed. The data are shown in Table 1.

**Table 1:** Sociodemographic and clinical analysis (*N* = 324)

Feature		Values (%)
Sex	Female	162 (50.00)
	Male	162 (50.00)
Age	Up to 60 years	149 (45.99)
	Over 60 years	175 (54.01)
Place of residence	Urban area	223 (68.83)
	Rural area	99 (30.56)
	No data available	2 (0.62)
Education	Primary	43 (13.27)
	Vocational	111 (34.26)
	Secondary	112 (34.57)
	Tertiary	58 (17.90)
Source of income	Employed	121 (37.35)
	Unemployed	36 (11.11)
	Retired	123 (37.96)
	Retired on ill-health grounds	44 (13.58)
Duration of diabetes	Up to 5 years	123 (37.96)
	5–10 years	80 (24.69)
	Over 10 years	120 (37.04)
	No data available	(0.31)

### Assessment of the level of self-care with SCODI questionnaire

The results of the SCODI questionnaire showed that the respondents scored the highest on adherence to recommendations concerning self-care maintenance, with an average of 68.37 points (SD = 18.14, Me = 68.75), and the lowest on self-care monitoring, with an average of 56.05 points (SD = 22.45, Me = 55.56). The data are shown in Table 2.

**Table 2:** Results of the SCODI questionnaire

SCODI	N	M	SD	Me	Min	Max	Q1	Q3
Self-care maintenance	324	68.37	18.14	68.75	12.5	139.58	56.25	81.25
Self-care management	324	63.85	24.34	64.71	0	211.76	50	82.35
Self-care monitoring	324	56.05	22.45	55,56	0	240.62	43.75	67.19
Self-care confidence	324	64.45	20.38	63.64	4.55	100	52.27	79.55

*SCODI* Self-Care of Diabetes Index, *N* number of patients, *M* mean, *SD* standard deviation, *Me* median, *Min* minimum value, *Max* maximum value, *Q1* quartile 1st, *Q3* quartile 3rd

### Assessment of adherence to treatment using the ACDS questionnaire

The assessment of adherence to treatment showed that 52.47% of the respondents presented a low level of adherence, 39.20%—a moderate level of adherence and only 8.33%—a high level of adherence. The data are shown in Table 3.

**Table 3:** Results of the ACDS questionnaire

ACDS—score	Interpretation	*N*	%
0–20	Low level of adherence	170	52.47%
21–26	Moderate level of adherence	127	39.20%
27–28	High level of adherence	27	8.33%

*ACDS* Adherence in Chronic Diseases Scale, *N* number of patients

### Correlation between adherence to treatment and the level of self-care

**Fig. 1:**
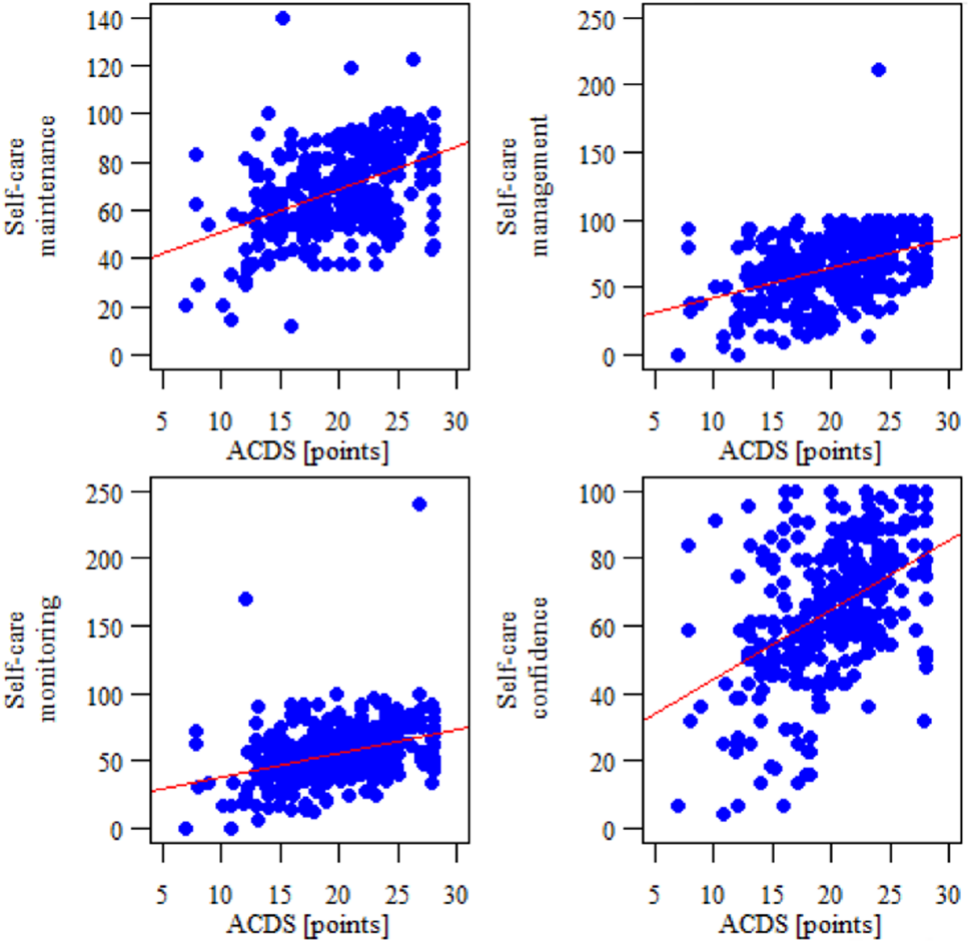
Correlation between the level of adherence and self-care ability

There was a significant positive correlation between the ACDS questionnaire scores and each subscale of the SCODI questionnaire (*p* < 0.05). Therefore, the higher the level of adherence to treatment, the higher the level of self-efficacy in each of the areas. The data are shown in Table 4 and Fig. 1.

**Table 4:** Correlation between the level of adherence and self-care ability

SCODI	Correlation with the ACDS			
	Correlation coefficient	p*	Direction of correlation	Strength of correlation
Self-care maintenance	0.436	*p* < 0.001 NP	Positive	Weak
Self-care management	0.413	*p* < 0.001 NP	Positive	Weak
Self-care monitoring	0.384	*p* < 0.001 NP	Positive	Weak
Self-care confidence	0.453	*p* < 0.001 NP	Positive	Weak

^*^P = Normal (parametric) distribution of both the correlated variables, Pearson correlation coefficient; NP = Non-parametric distribution (lack of normal distribution) in the case of at least one of the correlated variables, Spearman’s correlation coefficient

*SCODI* Self-Care of Diabetes Index, *ACDS* Adherence in Chronic Diseases Scale, *N* number of patients, *p* level of statistical significance

### Multiple factor analyses of the SCODI questionnaire

*“Self-care maintenance” subscale.* The linear regression model showed that the independent predictors of self-care maintenance were the following variables (*p* < 0.05): (1) ACDS score/adherence outcome/adherence ability: each additional point scored in the ACDS questionnaire increased the subscale score by an average of 1.476 points; (2) Sex: compared with female respondents, the subscale score for male respondents was lower by an average of 8.869 points; (3) Education: compared with respondents with primary education, the score for respondents with tertiary education was higher by an average of 9.241 points. The R^2^ coefficient yielded the level of 31.23%. The data are shown in Table 5a.

**Table 5:** Multiple factor analysis of the “Self-care maintenance”, “Self-care management”, “Self-care monitoring” and “Self-care confidence” subscales of the SCODI questionnaire

Variable		*R*	95% CI		*p*	*R*	95% CI		*p*
		“Self-care maintenance”				“Self-care management”			
(a)									
	ACDS [points]	1.476	1.088	1.864	< 0.001	1.797	1.271	2.324	< 0.001
Sex	Female	ref				ref			
	Male	− 8.869	− 12.457	− 5.28	< 0.001	− 5.3	− 10.176	− 0.424	0.033
Age	Up to 60 years	ref				ref			
	Over 60 years	− 0.547	− 6.024	4.931	0.844	− 6.452	− 13.894	0.991	0.089
Place of residence	Urban area	ref				ref			
	Rural area	− 1.708	-5.751	2.336	0.407	0.415	− 5.079	5.909	0.882
Education	Primary	ref				ref			
	Vocational	3.935	− 1.936	9.806	0.188	10.716	2.738	18.693	0.009
	Secondary	4.911	− 1.222	11.044	0.116	10.399	2.066	18.732	0.015
	Tertiary	9.241	1.873	16.61	0.014	16.554	6.543	26.565	0.001
Source of income	Employed	ref				ref			
	Unemployed	3.702	− 2.4	9.803	0.233	11.865	3.575	20.155	0.005
	Retired	2.039	− 3.892	7.97	0.499	4.681	− 3.378	12.74	0.254
	Retired on ill-health grounds	− 1.836	-8.133	4.462	0.567	− 8.844	− 17.401	− 0.288	0.043
Duration of diabetes	Up to 5 years	ref				ref			
	5–10 years	− 0.653	-5.301	3.994	0.782	− 0.727	− 7.042	5.587	0.821
	Over 10 years	1.017	− 3.581	5.615	0.664	3.31	− 2.937	9.557	0.298

(b)									
	ACDS [points]	1.59	1.073	2.106	< 0.001	1.834	1.401	2.268	< 0.001
Sex	Female	ref				ref			
	Male	1.287	− 3.493	6.068	0.597	− 0.47	− 4.481	3.541	0.818
Age	Up to 60 years	ref				ref			
	Over 60 years	− 1.883	− 9.179	5.414	0.612	− 2.944	− 9.066	3.179	0.345
Place of residence	Urban area	ref				ref			
	Rural area	4.846	− 0.541	10.232	0.078	0.685	− 3.835	5.204	0.766
	Primary	ref				ref			
Education	Vocational	4.439	− 3.383	12.26	0.265	7.64	1.077	14.202	0.023
	Secondary	11.02	2.85	19.19	0.008	9.962	3.107	16.817	0.005
	Tertiary	14.347	4.532	24.163	0.004	16.901	8.665	25.136	< 0.001
Source of income	Employed	ref				ref			
	Unemployed	4.758	− 3.37	12.886	0.25	7.902	1.082	14.721	0.023
	Retired	2.973	− 4.929	10.874	0.46	5.827	− 0.802	12.457	0.085
	Retired on ill-health grounds	− 5.659	− 14.048	2.73	0.185	− 4.833	− 11.872	2.206	0.178
Duration of diabetes	Up to 5 years	ref				ref			
	5–10 years	4.031	− 2.159	10.222	0.201	1.395	− 3.8	6.589	0.598
	Over 10 years	0.723	− 5.402	6.848	0.817	2.432	− 2.708	7.571	0.353

*SCODI* Self-Care of Diabetes Index, *ACDS* Adherence in Chronic Diseases Scale, *N* number of patients, *R* regression parameter, *CI* confidence interval, *p* level of statistical significance

*“Self-care management” subscale.* The linear regression model showed that the independent predictors of behaviours such as self-care management behaviours were the following variables (*p* < 0.05): (1) adherence factor (ACDS score): each additional point scored in the ACDS questionnaire increased the subscale score by an average of 1.797 points; (2) Sex: compared with female respondents, the subscale score for male respondents was lower by an average of 5.3 points; (3) Education: compared with respondents with primary education, the subscale score for respondents with vocational education was higher by an average of 10.716 points, the score for the respondents who had secondary education was higher by an average of 10.399 points and the score for respondents with tertiary education was higher by an average of 16.554 points; (4) Source of income: compared with the respondents who were employed, the subscale score for unemployed respondents was higher by an average of 11.865 points, and the score for the respondents who were retired on ill-health grounds was lower by an average of 8.844 points. The R^2^ coefficient was at the level of 29.84%. The data are shown in Table 5a.

*“Self-Care monitoring” subscale.* The linear regression model showed that the independent predictors of a score on Self-care monitoring were the following variables (*p* < 0.05): (1) adherence outcome/adherence ability: each additional point scored in the ACDS questionnaire increased the subscale score by an average of 1.59 points; (2) Education: compared with the respondents who had primary education, the score for respondents with secondary education was higher by an average of 11.02 points, and the score for respondents with tertiary education was higher by an average of 14.347 points. The R^2^ coefficient was at the level of 20.4%. The data are shown in Table 5b.

*“Self-care confidence” subscale. *The linear regression model showed that the independent predictors of self-care confidence were the following (*p* < 0.05): (1) ACDS score adherence outcome/adherence ability: each additional point scored in the ACDS questionnaire increased the subscale score by an average of 1.834 points; (2) Education: compared with the respondents who had primary education, the score for respondents with vocational education was higher by an average of 7.64 points, the score for respondents with secondary education was higher by an average of 9.962 points and the score for respondents with tertiary education was higher by an average of 16.901 points; (3) Source of income: compared with the respondents who were employed, the subscale score for unemployed respondents was higher by an average of 7.902 points. The R^2^ coefficient was at the level of 31.67%. The data are shown in Table 5b.

The R^2^ coefficient, i.e. the proportion of the variance of the results which is explained by the variables included in a given model, for the above models ranged from 20.40% (for self-care managemen) to 31.67% (for self-care confidence). Thus, the proportion of the variance which is explained by variables not included in the models and random factors ranged from 68.33% to 79.60%.

## Discussion

DM is one of the fastest growing diseases in the world. Despite the availability of treatment, patients do not manage to control the disease due to failure to comply with therapeutic, dietary and physical activity guidelines. Contemporary health care practice is to promote and support people with chronic illness to self-care management [18]. For that reasons, quantitative assessment how self-care affects adherence to treatment in patients diagnosed with type 2 DM becomes a very important for nursing practice. Identifying causes for poor or none of self-care behaviours, and non-adherence in DM patients, requires that relevant steps be taken to enhance the self-efficacy of patients and improve their treatment outcomes.

The existing studies suggest that patients diagnosed with DM have a moderate level of knowledge of their condition and that their level of knowledge depends mainly on such factors as age, education and duration of illness. It is necessary to provide patients with health education tailored to their needs. Healthcare workers play a major role in identifying problems and prepare suitable programmes on that basis [19]. The latest clinical recommendations of the Polish Diabetes Association show that blood glucose monitoring and analysis is an integral part of the management of DM. The document stresses how important is the systematic education of patients about self-monitoring, including education information about a glucose metre and ways of interpreting measurements regarding changes of diet, physical activity and medication doses [20]. We ran the study on a group of 324 patients diagnosed with type 2 DM. The patients scored the highest on adherence to self-care maintenance recommendations and the lowest on self-care monitoring. The literature mentions seven basic behaviours relating to diabetes self-care which are essential to good treatment outcomes. They include a healthy diet, physical activity, blood glucose monitoring, adherence to medication, ability to solve problems, ability to cope with stress as well as risk mitigating behaviours [21]. These behaviours were found to correlate positively with good blood glucose levels, reduction of complications, improved quality of life and improved adherence to treatment [22–24].

Adherence to treatment in chronic conditions is a significant issue raised in the recent literature. Our study found that 52.47% of the respondents showed the low level of adherence, 39.20% of patients adhered at the moderate level and only 8.33% of patients presented the high adherence to treatment, which confirms how serious the problem is Poland in terms of self-management activities. A similar study by Polonsky and Henry [25] carried out on patients with DM found that only 30% of study participants adhered to treatment regimens and that patients with lower socio-economic status showed a lower level of adherence. In other study by García et al. [26] on the management of DM and adherence to treatment showed that self-efficacy was a significant predictor of later adherence to treatment of DM. Based on a cross-sectional study conducted among 419 patients with type 2 diabetes, Bonger et al. [10], showed a lack of adherence to dietary recommendations in more than 75% of the respondents. As many as 83.5 patients did not adhere to self-monitoring of blood glucose level and almost 20% were not taking the prescribed medications.

The other study by Shrivastava et al. [5] stressed the importance of both patient-related factors, such as adherence to recommendations, proactive attitude, convictions, knowledge of diabetes, financial resources, co-morbidities and social support, and factors relating to medical teams, such as effective communication, knowledge and treatment planning skills, as factors which determine the success of treatment.

Our study demonstrates the importance of sociodemographic factors in managing type 2 DM. We found that female patients, patients with tertiary education and unemployed patients showed a higher level of self-care behaviours. Bonger et al. [10] investigated self-care in 419 patients with type 2 DM and found that 75.9% of the patients failed to adhere to dietary recommendations, while 83.5% of the patient sample did not monitor their blood glucose levels. Moreover, the study of Bonger et al. [10] showed that unemployed patients, patients with tertiary education and younger patients were more likely to adhere to treatment regimens. Tekalegn et al. [27] list the duration of the disease and the type of treatment used among the determinants influencing adherence to the therapeutic recommendations. In a group of over 400 patients, the predictors of worse glycemic control were longer disease duration and insulin therapy.

Recognising the multidimensional nature of the problem, systematic and multifaceted approach must be taken to improve the level of self-care in patients with DM to improve adherence and the effectiveness of treatment. Achieving high levels of therapeutic adherence in diabetic patients should be a key objective for health-care workers. The available literature also suggests, that new and innovative approaches need to be encouraged, clinically tested, and then implemented.

Accoridng to the implications for clinical practice*,* it is recommended that a routine assessment of the level of self-care, including adherence, be carried out in patients diagnosed with type 2 DM. Early identification of knowledge deficits about self-care is of vital importance for the provision of health education tailored to patients’ needs. Improved adherence to treatment in patients with type 2 DM may help prevent or delay complications of the condition. The study clearly indicates the importance and role of self-care in achieving a satisfactory level of the adherence with the therapeutic recommendations. There is a need to pay attention to the phenomenon of self-care and self-management in the group of patients with type 2 diabetes. The assessment of the quality of care in the discussed population seems to be important. Such actions will allow monitoring of patients' self-management [28]. The use of health education is an important tool to support and promoting behaviours that contribute to improving self-care among type 2 diabetes patients [8]. Health education should therefore be an integral part of clinical and outpatient practice to support patients with type 2 diabetes.

## Conclusions

The self-care management behaviours have a significant impact on adherence to treatment in patients with type 2 DM. The higher the level of self-efficacy in each of the areas of functioning, the higher adherence. It was found that female patients, patients with tertiary education and unemployed patients showed a higher level of type 2 DM self-care.

### Limitations of the study

The results of the study clearly indicate the importance of self-care for adherence in the group of patients with type 2 diabetes. The limitation of the study was the lack of analysis of clinical parameters (e.g. blood glucose and therapeutic haemoglobin (HbA1c) levels) indicating adherence to therapeutic recommendations and their correlation with the domains of the self-care questionnaire. It seems necessary to conduct further research using health education and to assess its impact on adherence and selfare.

## Data Availability

The datasets generated during and/or analysed during the current study are available from the corresponding author on reasonable request.
